# Consumer Trust in Food and the Food System: A Critical Review

**DOI:** 10.3390/foods10102490

**Published:** 2021-10-18

**Authors:** Wen Wu, Airong Zhang, Rieks Dekker van Klinken, Peggy Schrobback, Jane Marie Muller

**Affiliations:** 1 Data61, The Commonwealth Scientific and Industrial Research Organisation (CSIRO), Brisbane 4102, Australia; 2 Health and Biosecurity, The Commonwealth Scientific and Industrial Research Organisation (CSIRO), Brisbane 4102, Australia; airong.zhang@csiro.au (A.Z.); rieks.vanklinken@csiro.au (R.D.v.K.); jane.muller@csiro.au (J.M.M.); 3 Agriculture and Food, The Commonwealth Scientific and Industrial Research Organisation (CSIRO), Brisbane 4067, Australia; peggy.schrobback@csiro.au

**Keywords:** assurance, food actor, packaging label, traceability, supply chain operator, food industry influencer

## Abstract

Increased focus towards food safety and quality is reshaping food purchasing decisions around the world. Although some food attributes are visible, many of the attributes that consumers seek and are willing to pay a price premium for are not. Consequently, consumers rely on trusted cues and information to help them verify the food quality and credence attributes they seek. In this study, we synthesise the findings from previous research to generate a framework illustrating the key trust influencing factors that are beyond visual and brand-related cues. Our framework identifies that consumer trust in food and the food system is established through the assurances related to individual food products and the actors of the food system. Specifically, product assurance builds consumer trust through food packaging labels communicating food attribute claims, certifications, country or region of origin, and food traceability information. In addition, producers, processors, and retailers provide consumers with food safety and quality assurances, while government agencies, third-party institutions, advocacy groups, and the mass media may modify how labelling information and food operators are perceived by consumers. We hope our framework will guide future research efforts to test these trust factors in various consumer and market settings.

## 1. Introduction

The globalisation of food supply chains and the increasing complexity of modern food systems are changing the relationship consumers have with food. Enhanced focus towards food safety and quality has reshaped the way contemporary consumers evaluate food and make purchase decisions. Specifically, several serious food safety incidents, cases of food fraud, and changes in food production practices have violated consumer trust across the globe [1,2,3,4,5]. While not all of these incidents have directly imposed risks to public health and safety, they do represent a breach of consumer trust and have reduced consumer confidence in the integrity of the food system [1,2,6,7,8,9].

As food supply chains become increasingly globalised, there is a need to understand the range of cues and information that consumers trust and rely on when navigating the complexities of the modern food system. Consumers with high levels of trust have confidence in the quality of the food items they are purchasing, and that the food operators who produce, distribute, and sell food are meeting relevant standards [10]. Food quality refers to the tangible sensory characteristics of food, such as taste, flavour, freshness, juiciness, and appearance [11]. Food quality can also incorporate less tangible credence attributes including nutritional value, functional quality, and convenience aspect of food, as well as ethical and environmental considerations. In some instances, food quality represents prestige, and an individual’s social status and wealth [12,13] Food safety can also be considered as an attribute of food quality, and it refers to the safe handling and storing of food [14,15]. Safe foods are free from harmful biological, chemical, or physical agents.

Growth in income and awareness of the health, social, and environmental consequences of food choices has increased global demand for diverse, safe, and premium foods with specific attributes (e.g., organic, eco-friendly, nutritious) [16,17,18,19,20]. Consumers commonly use direct visual and physical cues related to individual food items (e.g., colour, firmness, size, and price) when assessing food quality. For example, consumers from Asia tend to assess meat quality based on the colour of the meat and its level of intramuscular fat [21,22]. However, there are a growing number of credence attributes that consumers consider in their purchasing decisions that cannot be directly inferred through search or experience, such as safety, nutrition, environmental protection, and animal welfare. Traditionally, branding, marketing, and advertising have been used to communicate such credence attributes of certain food items and influence consumer choice [23,24]. Over the past decades, however, the growing complexity and globalised scale of the food system have presented increased opportunities for fraud and food safety incidents to occur [25,26]. Consequently, consumers must rely on additional cues and information to help them assess the full suite of credence attributes they seek [16]. Thus, the scope of this review is focused towards outlining the cues and information beyond product branding and marketing that consumers trust for verifying the less visible food attributes.

Extensive research has examined various factors that influence consumer trust, including trust in supply chain actors, assurance and regulatory systems, and the economic development status of the producing country [14,16,17,27,28,29,30]. While these efforts have advanced our understanding of consumer trust, much of the previous studies have focused on particular aspects of trust in food and the food system, with some findings being country and culture specific [31,32]. As a result, the factors influencing consumer trust identified in the literature are often fragmented, and at times, contradictory.

In this study, we critically review the literature to generate a framework showing the diverse range of factors that influence consumer trust in food and the food system. We use this framework and review to discuss the trust-influencing factors that are well-supported by previous research, as well as areas that future research could further explore.

## 2. Materials and Methods

**Figure 1:**
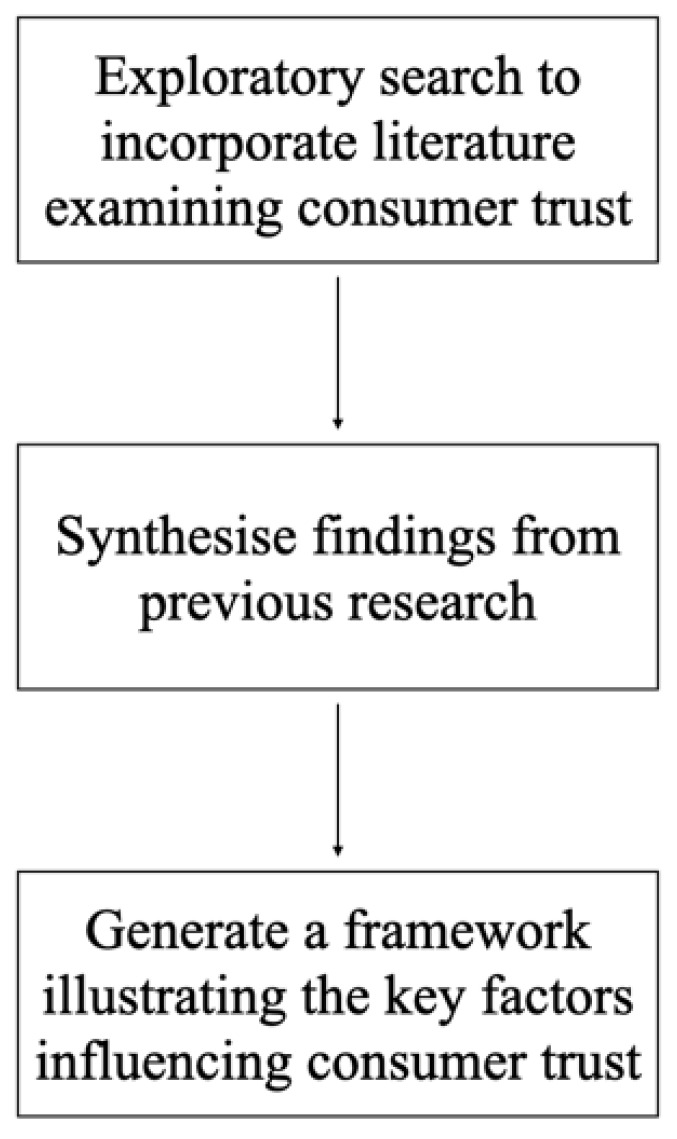
A flowchart illustrating the methodology steps.

Given the vast amount of existing research examining consumer trust, we aim to critically synthesise the main themes discussed in the literature and construct a framework outlining the key factors influencing consumer trust (Figure 1).

An exploratory search was conducted to incorporate literature examining trust indicators used by consumers, food-related attributes that attract a price premium, consumer trust in the food supply chain, consumer trust in product credentials and verification systems, and consumer use of food verification information. The selection criteria for studies to be considered in our review include that the study is written in English, the study is published in a peer-reviewed journal, and the study is published between 2000 and 2021.

For the literature search, we used search terms related to ‘consumer trust’, ‘purchasing behaviour’, and ‘trusted food indicators’ to explore the key attributes that consumers look for and rely on when purchasing food items to ensure that the food is safe and of high quality. Search terms related to ‘willingness to pay’ for food with added attributes were included as well. In addition, ‘trust in food certification’, ‘trust in food traceability systems’, and ‘valued information used to verify food credentials’ were used to evaluate what information consumers rely on when verifying food attributes. Lastly, terms related to ‘trust in the food supply chain’ were used to support the analysis of consumer trust in supply chain actors. The search terms were applied as keywords in the titles, abstracts, and body of the journal articles in academic databases, including Emerald Insights, PLoS ONE, MDPI, Wiley Online Library, Springer Link, ProQuest, Taylor and Francis Online, Oxford Academic, and ScienceDirect.

Our search identified 133 relevant articles examining factors influencing consumer trust in food and the food system. Of the reviewed journal articles, approximately 40 percent evaluated trust in domestic foods, 40 percent examined both domestic and imported foods, and 10 percent assessed imported foods only. Food categories investigated in these studies included liquid milk, infant milk powder, eggs, cheese, meats, organic food, fruit, vegetables, rice, cooking oil, Fairtrade products, and sustainable foods. Because this review aims to summarise the range of factors shaping consumer trust, we took the consumer perspective and only included articles that investigated cues and information that are visible to the consumer.

## 3. Results and Discussion

**Figure 2:**
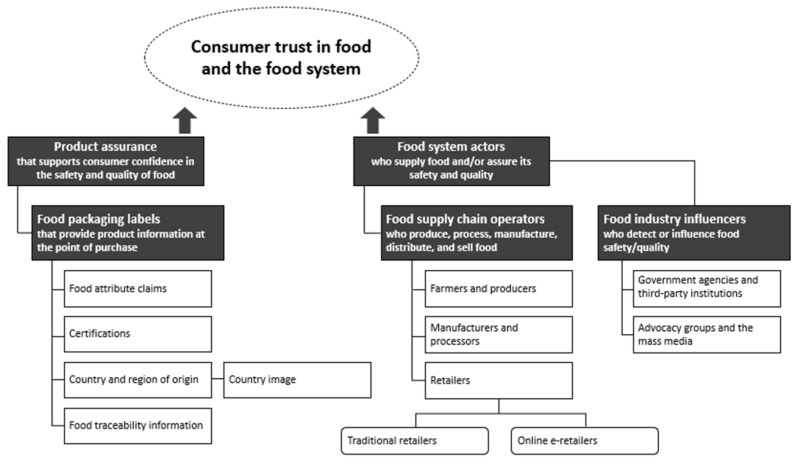
A framework summarising the range of factors that influence consumer trust in food and the food system.

We developed a framework that summarises the range of diverse trust-influencing factors reported in the literature that help consumers verify the less visible food attributes they seek (Figure 2). Our first step was to list all the factors, identified from our review, that influence consumer trust in food and the food system. We then grouped the different factors according to how they are perceived by the consumer and incorporated them into the framework.

Our review showed that the identified factors influence consumer trust in two distinct ways: either at the product level through labelling, or indirectly through actors of the food system. Providing direct assurance on the safety and quality of food items through food attribute claims, certifications, country and region of origin, and food traceability information, builds consumer confidence and trust. Because these factors related to product assurance are mainly made accessible to consumers through printed labels on food packaging, they were summarised in the framework as factors related to food packaging labels that consumers trust. Our description of food packaging labels also incorporates product related information that appear on information boards in physical retail stores, especially for nonpacked foods, or on a webpage of an online retail store. In addition, we found two separate groups of food system actors who play a role in influencing consumer trust. While consumers trust producers, processors, and retailers for supplying high quality foods, government agencies, third-party institutions, advocacy groups, and the mass media are positioned to modify how labelling information and food operators are assessed by consumers.

We present the key factors related to product assurance and food system actors that influence consumer trust. Differences in findings are also discussed from the perspectives of culture and economic development.

### 3.1. Product Assurance

Product assurance aims to enhance consumer confidence in food and demonstrate that it is authentic and meets relevant industry standards for safety and quality. Because food items are often accompanied with printed labels that describe various credence attributes that consumers are seeking, food packaging labels are the primary tangible cues that consumers have access to and use at the time of purchase to validate product attributes and assurance claims (Figure 2).

#### Food Packaging Labels

Product labelling is the written information on the packaging of a food product that informs consumers about its unique attributes, quality certifications, country of origin, and production region. It also enables access to food traceability information. Labelling information, and links to traceability information, can also be presented on information boards in physical retail stores, as well as appear on a webpage when consumers are shopping online. Labels act as a direct communication channel from producers, retailers, regulators, and third-party certifiers to consumers [33]. They can also display branding and marketing information, food safety and quality information, as well as information including ingredients, instruction, and uses. Food packaging labels, therefore, are positioned at the interface between consumers, food, and the food system. From our analysis of the various factors influencing consumer trust, we consider food packaging labels that communicate food attribute claims, certifications, country and region of origin, and food traceability information to be the most tangible information sources that consumers can rely on for identifying and validating the different credence attributes they seek.

*Food attribute claims.* Food attribute claims mostly include quality related characteristics that cannot be visually or physically examined by the consumer at the time of purchase. These quality characteristics include, for example, taste, ingredients, and production methods. Ethical and environmental considerations, as well as the nutritional value, functional quality, and convenience aspects of food, among others, are also less tangible and require labelling for consumers to distinguish. While food attribute labels can include a variety of information and vary from product to product, research shows that consumers tend to prefer simple and clearly presented food attributes on packaging, such as ‘free-from’, ingredients, nutritional information, and expiry date [16,17,34,35,36,37]. For example, Australian consumers tend to look for simple labels describing the specific cut of meat to help assess its eating quality. Asian consumers, on the other hand, tend to value a variety of different labelling information, including country and region of origin, attributes associated with improved health, and the personal quality of producers [37].

*Certifications.* Food labels can also display certification information that supports specific credence claims that are otherwise un-observable, such as organic, Halal, free-range, animal welfare, environmental sustainability, or Fairtrade. Certification labels help consumers validate authenticity and support their confidence in food. Studies of certification labelling show that scientifically certified expert labels are the most trusted. A study of 10,000 consumers from Japan, the United States, Germany, China, and Thailand showed that consumers trusted certified labels endorsed by scientific experts more than those supported by producers, the government, and consumers [38]. Trust in scientific experts indicates the value of scientific testing for food quality and safety assurance.

Increased food scares and incidents across many developing countries have motivated consumers from these regions to trust international certifications more than domestic equivalents [14,35,39,40,41,42,43]. However, there is contradictory evidence suggesting that some consumers can be discouraged from purchasing internationally certified food products [22,27,29,42,44,45]. These findings could relate to consumers in developing countries being less familiar with international certification labels, or perceiving offshore certification as more costly than domestic certification [22,27,29,42,44]. Another variation to the general preference in developing countries for international certification is associated with the certification of foods related to a specific cultural domain, such as Halal. For example, Malaysian consumers tend to prefer and trust Halal certified food products from Muslim countries, such as Saudi Arabia, Pakistan or Brunei, over certifications from non-Muslim countries such as China, New Zealand, European countries, and Thailand [45].

*Country and region of origin.* Geographical labels are often used as an important differentiating tool for assessing food safety, quality, and authenticity [46]. Specifically, consumers use geographical labels as the main differentiation tool for high-value products, such as wine [46]. Moreover, geographical labels are sometimes presented alongside other attributes, such as eco labels, for consumers to develop brand associations [47]. The literature indicates that geographical labels are valued by all consumer segments regardless of culture [21,37,48].

*Country image.* Globalisation of the food industry enables consumers to access a diverse range of foods from countries and regions around the world. Studies indicate that consumers often rely on their general perception, or the country image, of the producing country as an indication of food safety and quality [49,50]. The perceived ‘image’ or overall impression a consumer has about a country creates a ‘halo effect’ of positive or negative feelings towards products that are produced from that country or place [14,51,52,53]. 

In general, positive feelings about an exporting country being warm and friendly build trust [49,54,55]. Positive feelings derived from a perceived ‘clean and green’ image of an exporting country also play a role in influencing consumer trust. The degree to which a country is perceived to have a naturally beautiful and unspoiled environment has been shown to increase consumers’ willingness to purchase imported foods from that country [49,56]. For example, consumers in France perceived eco-labelled products less favourably when associated with a country that tends to portray less of an eco-friendly image than when the same products were associated with a country perceived as environmentally friendly [57]. 

The economic development of a country, and its perceived competence, also act as an important cue for food safety and quality [17,28,49]. In general, food safety incidents across Asia have resulted in many consumers preferring imported foods from economically developed countries over domestic foods. For example, consumers in China rated genetically modified orange juice coming from Australia and the United States as higher quality than genetically modified juice from China, Brazil, and Israel [58]. Increased trust towards economically developed countries for producing genetically engineered foods seems to suggest an existing perception among consumers that these countries have better regulatory systems in place, and are more advanced in implementing agricultural technologies and biotechnologies [58]. 

In contrast, European consumers prefer food products from countries that are geographically closer in distance [29,59]. Even in Albania and Kosovo where food safety incidents related to domestic production are prevalent, consumers still consider domestic food to be safer and of higher quality than imported foods [34]. A study of consumer preference for the origin of seafood in Europe showed consumers to value domestic, local, and European production over foreign imports [60]. In addition, consumers in Denmark consider geographical origin as more important than whether or not the food is organic or the size of the production company [61]. European consumers believe that locally produced foods are fresher, require less transport, and are better regulated than foreign foods [62]. They also value the economic benefits associated with purchasing locally produced foods [61]. Across other developed economies, similar domestic and local preference has been found among consumers in the United States and in Australia [63,64].

*Food traceability information.* Food traceability systems capture information related to the origin of food products and document their journey across the supply chain [3]. These systems play an important role in supporting food safety and quality by providing increased transparency across the food supply chain [65]. While food traceability has historically been used as a supply chain risk management tool by agribusinesses and retailers, it is increasingly applied to enhance consumer confidence in food authenticity, safety, and quality. Traceability data is made accessible to consumers through barcodes, Quick Response (QR) codes, radio-frequency identification, and online links printed on food packaging [3,66,67]. Supported by cloud computing, storage technologies, and more recently decentralised blockchain networks, food traceability information made available to consumers reduces information asymmetry and increases trust [3,68,69,70]. Food traceability systems offer consumers reassurances on the origin and history of food products, raise the standards of food safety and quality across the international markets, and help build consumer confidence and trust in the traced food products [71,72,73,74].

Traceability information that verifies where the food product was produced, and how it was processed and grown, reassures consumers about the food’s authenticity, and provides substantial value in assisting the management of food incidents [8,75,76,77,78,79,80,81,82,83]. Across the developing countries, increased food safety instances and fraud have intensified consumer demand for detailed information about origin, manufacturing processes, and agricultural inputs [72,84,85,86,87,88,89]. Studies show that consumers from China, Vietnam, Malaysia, and Brazil value food traceability and are willing to pay a price premium for traced foods [37,44,72,90,91,92,93,94,95]. 

However, in developed countries where there have been fewer food safety incidents, studies show consumers engage less with food traceability information. Interviews with the major Australian retailers revealed that time-poor consumers often have little interest in the mechanics of the food supply chain [68]. Others have found that, while Australian consumers put more emphasis on having imported food traced, their trust in the information provided by the traceability system of imported food products was low [96]. Thus, more work may be needed to establish strategies to increase consumer knowledge about food traceability systems across the globe and their engagement with the tracked data [96,97].

While traceability systems designed to improve consumer confidence through increased transparency are promising, consumers trust in traceability systems and information is less clear. Emerging evidence suggests that traceability information that is validated by an independent party helps to build consumer trust. Through a discrete choice experiment with Fuji apple in China, Liu et al. (2020) found that the majority of Chinese consumers regarded traceability validation as far more important than the traceability itself. In addition, they placed the highest value on government validation and were willing to pay a price premium for apples validated by the government compared to those validated by domestic or international third-party certifiers.

### 3.2. Food System Actors

While providing product assurance through food packaging labels directly supports consumer confidence in food products, food system actors also play an important role in influencing consumer confidence and trust in food and the food system. Food system actors are those who are directly involved in the production and distribution of food, including farmers and producers, manufactures and processors, and retailers. On a day-to-day basis, consumers have the greatest opportunity to develop face-to-face reciprocal relationships with retailers. Their engagement with the upstream food operators, such as producers and food companies, tends to be minimal. In the event of food incidents, however, upstream food actors often become more of a focus for consumers [88,98,99]. Food system actors also include industry influencers who assure or monitor food safety and quality, including government agencies and third-party institutions, consumer advocacy groups, and the mass media. Although industry influencers are not directly responsible for the production and distribution of food, they are positioned to influence how food is governed and certified, and can directly communicate with the public about food safety and quality. In the remaining of the Results and Discussions section, we outline the actors along the supply chain, including food industry influencers, to whom consumers trust and assign responsibility for ensuring food safety and quality.

#### 3.2.1. Food Supply Chain Operators

Globalisation and the complexity of the food supply chain is increasing the physical distance between consumers and industry operators who produce, process, manufacture, distribute, and sell food [25,100]. Consequently, consumers often have limited knowledge about where their food has been grown and produced, and how it has been processed and distributed along the supply chain [98,101]. Research suggests that consumers tend to put more trust in the operators from the downstream parts of the food supply chain, such as retailers, than those from upstream [48].

*Farmers and producers.* Consumer trust in the supply chain operators who directly grow and produce food differs around the world. Consumers from countries that experience limited food safety scares and rely on local produce tend to put more trust in famers [98,102]. For example, Henderson et al. (2011) found that consumers in Australia display high levels of trust towards farmers. Similarly, consumers across Europe, especially those from France, Poland, and Italy, tend to base their purchasing decisions on the farmer who produced the food [31,103]. Consumers from developing countries, on the other hand, tend to trust farmers the least. For example, food scares and fraudulent practices across many cities of China have reduced trust among urban consumers in local farmers and producers [38,104]. However, consumers from rural China who are able to source food directly from farmers tend to put more trust in local farmers who also produce for their own consumption [27,32].

*Manufactures and processors.* In general, consumers’ connection with and trust towards food manufactures and processors tends to be low [3,31]. During food safety incidents, consumers tend to direct the responsibility for re-building and maintaining trust towards these actors. An online survey of consumers in France, Germany, Poland, Spain, and the United Kingdom showed that trust is established by consumers’ belief that supply chain actors, especially manufactures, are competent, caring, and open [31]. Consumer trust in domestic food manufactures tends to be low in China [42]. However, there are indications that the introduction of new food safety laws, following food product scandals, has improved some Chinese consumers’ confidence and trust in manufactures for ensuring food safety and quality [105].

*Retailers.* As consumers become more disconnected from food supply chain operators, especially those from the upstream producers, processors, and manufactures, they increasingly rely on retailers to ensure food safety and quality. Both traditional and online retailers build trust through developing ongoing consumer–retailer relationships, ensuring all economic transactions are secure, and fostering positive consumer feedback [106,107,108,109]. Traditional retailers with a physical presence, such as supermarkets, grocery stores, butchers, and farmers’ markets, build trust through direct consumer–retailer relationships and food networks [3]. Personal trust developed between the consumer and retailer acts as a cue for food quality and safety [34]. For example, consumers from Albania indicated that their main source of trust when evaluating food quality and safety is knowing the butcher or seller [110]. Across China, where food fraud and safety incidents are common, consumers reported the highest level of trust in large reputable international retailers selling imported foods from developed countries [27,32,104]. Despite a price premium on imported foods from reputable stores, these retailers are perceived to offer greater assurances on product authenticity and operate within the regulatory control of the government [37,111]. Chinese consumers tend to perceive smaller independent retailers and street vendors as more risky and likely to be involved in fraudulent practices [32].

Compared to traditional face-to-face retailers who physically operate in a commercial location, consumer trust in online e-retailers can be more fragile and difficult to establish [112]. E-retailers need to use more indirect approaches to build trust with their customers, such as the look of their website and online reviews. A study of Chinese consumers found organic food information presented on a media-rich website reduced perceived risk and improved trust in the retailer [113]. Another study of 420 mobile shopping app users in India showed that online trust can be highly influenced by the visual attractiveness of the mobile shopping app [114]. Innovative website features have also been shown to build trust towards online shopping sites and increase consumers’ repurchasing intentions [109]. In particular, e-retailer sites with added human and social elements strengthened trust through providing consumers with an indirect sense of personalness and warmth [115,116,117]. Online reviews also enable consumers to indirectly access product quality and safety information through the lived experiences of other consumers [113]. Long reviews with more detailed information of a product are perceived as higher quality and more useful than simpler reviews [118]. In addition, positive or factual reviews, and reviews appearing on social networks, are often perceived as more trustworthy than negative or emotional reviews, and reviews appearing on retailer sites [118]. 

#### 3.2.2. Food Industry Influencers

Food industry influencers are in a position to confirm or challenge the legitimacy of the food attribute claims made by food actors, and to shape consumers’ perception of the risks related to food safety and quality. While government agencies and third-party institutions influence consumer trust through certification labelling that validates certain food attribute claims, consumer advocacy groups and the mass media influence trust through directly communicating with consumers about the trustworthiness of food operators. There are some minor cultural differences in who consumers trust for communicating the relevant information relating to food safety and quality. 

*Government agencies and third-party institutions.* Government regulatory agencies and third-party institutions can enhance consumer confidence through providing independent validation and certification of the credence attributes claimed by food producers, processors, and manufactures. The validation provided by these influencers can be communicated to consumers through product labelling. As a consequence of increased food incidents across many developing countries, consumers from these regions trust government authorities and third-party institutions, over food supply chain operators, for ensuring the safety and quality of domestically produced food products [111,119]. For example, consumers in Taiwan display higher levels of trust and preference towards government-supported food inspections over those conducted by operators [119]. Chinese consumers hold high levels of trust towards independent institutions for providing quality and safety guarantees of domestically produced foods [105]. They generally perceive government and third-party institutions (e.g., research institutes and consumer associations) to be knowledgeable and accurate in their communication of food safety and quality information, and care about the health and wellbeing of the public [111,119].

*Advocacy groups and the mass media.* As consumer demand for ethical food production and consumption increases across the developed countries, advocacy groups who communicate directly with consumers about the trustworthiness of food operators for producing ethical foods are playing an increasing role in influencing consumer trust [120]. The mass media, in particular traditional news channels and social media, has been an important portal for advocacy groups to rapidly expose poor behind-the-scenes practices, food fraud, and food safety incidents [32,111,119]. Media representation can powerfully influence consumer perceptions of food-related issues, especially those with which the general public have limited direct experience [120]. For example, animal welfare activists exposed industry wrong doings within the live export industry through broadcasting video footage of animal mistreatment in the slaughterhouse of the importing countries, and poor welfare conditions within intensive piggeries and broiler sheds [120]. The widespread broadcast of these videos across the mass media led to the suspension of Australian cattle exports to countries exposed for mistreating animals and the closure of Australian operations with poor animal welfare. The work of advocacy groups has given rise to dramatic changes in consumer attitudes towards animal production and welfare [121]. Bray and Ankeny (2017) found that Australian consumers perceive free-range and cage-free eggs as better quality, more nutritious, safer, and tastier than caged eggs. These changes have accelerated consumer demand for ethical foods and have pushed many retailers across the developed countries to establish new animal welfare standards for producers [120].

## 4. Conclusions

Income growth, increased awareness of health benefits of foods, and ongoing food safety incidents are accelerating consumer demand for safe and quality foods that cannot easily be distinguished based on visual and physical cues. Although branding and marketing strategies are widely used to communicate the credence attributes of food products, the complex, fragmented, and globalised modern food system presents growing opportunities for food incidents, fraud, and poor practices to occur. Consequently, agribusinesses and supply chain stakeholders have taken various approaches to address consumer concerns and secure their confidence in food safety and quality.

The present review developed a food trust framework and revealed that consumer trust can be built through product assurance and food system actors. Our findings show that product assurance through food packaging labels that communicate food attributes, certifications, country or region of origin, and food traceability are one important set of tangible cues and sources of information that consumers trust when assessing food safety and quality at the time of purchase. In addition, food system actors, particularly retailers and food industry influencers, who detect or directly communicate with the public about food safety and quality, also play a crucial role in influencing consumer trust.

While there are similarities in the cues and information consumers trust and rely on for ensuring food safety and quality across the globe, the specific ways in which these factors operate can vary between countries and cultures. For example, geographical labels indicating the country and region from which the food was produced tend to be valued by all consumers. However, consumers from Europe tend to primarily rely on geographical origin for evaluating food safety and quality, while consumers in Asia tend to rely on more detailed labelling information that includes origin, but also health related attributes and personal qualities of producers. 

Ongoing food safety incidents seem to have motivated consumers across developing countries to prefer imported foods and quality certifications from economically developed countries over domestic equivalents. Despite a price premium on foods, consumers from developing countries appear to trust reputable international retailers and manufactures over local producers. They tend to trust government authorities and third-party institutes for regulating and validating food safety and quality over advocacy groups and the mass media. In many of the developed countries, however, there is a general preference for domestic food products over foreign imports, even in countries where food safety incidents related to domestic production are prevalent. Consumers from developed countries with fewer experiences of food incidents tend to put more trust in local farmers and retailers who sell local produce than food manufactures. Compared to consumers from developing countries, the purchasing decisions of those from developed countries tend to be influenced more by the advocacy movements championing animal warfare and ethical consumption.

Our findings also suggest that consumers may have greater confidence in product assurance systems that are supported by the food system actors they trust. While food packaging labels help consumers validate credence attributes, food system actors provide consumers with an added layer of safety and quality assurance. To contrast consumers from developed countries, those from developing countries tend to trust government authorities and third-party institutions (e.g., research institutes and consumer associations) over food supply chain operators for validating food traceability information and ensuring the safety and quality of domestic food products. While digital and traceability systems present opportunities for food system actors to provide consumers with added transparency and assurance across the food supply chain, research investigating consumer trust in traceability information appears limited at present. Future research needs to establish a better understanding of what traceability information consumers value and the validation that they trust. It should also be noted that there appears to be a disconnect between what consumers state they trust under experimental settings and their actual purchase behaviour at the time of purchase [23]. Improved methods need to be developed that better capture actual consumer behaviour under experimental settings.

Overall, our framework offers the first step in understanding the key drivers of consumer trust and how those may be influenced by culture. While our framework highlights the individual importance of food packaging labels, supply chain actors, and industry influencers when evaluating consumer trust, more work is required to establish their relative importance. Knowing the relative importance of these main factors influencing consumer trust has implications for the design and further development of credentialing and traceability systems. We hope our framework will guide future research and food industry efforts to test these trust factors in various consumer and market settings, and develop systems and strategies for building and maintaining consumer trust.

## Data Availability

Not applicable.
